# Differentiated Effects and Determinants of Home Blood Pressure Telemonitoring: Three-Year Cohort Study in Jieshou, Anhui, China

**DOI:** 10.2196/37648

**Published:** 2022-10-11

**Authors:** Qun Xue, Xuewu Zhang, Rong Liu, Xiaoqin Guan, Guocheng Li, Linhai Zhao, Qian Wang, Debin Wang, Xingrong Shen

**Affiliations:** 1 School of Health Service Management Anhui Medical University Hefei China; 2 Department of Public Health Jieshou Hospital Fuyang China

**Keywords:** blood pressure, home telemonitoring, effect, influence factors, China

## Abstract

**Background:**

Home blood pressure telemonitoring (HBPT) is witnessing rapid diffusion worldwide. Contemporary studies documented mainly short-term (6-12 months) effects of HBPT, and there are limited data about its uptake.

**Objective:**

The aim of this study was to explore the 3-year use and determinants of HBPT, and the interactions with systolic and diastolic blood pressure (SBP/DBP) and overall blood pressure (BP) control rate.

**Methods:**

HBPT records were obtained from a 3-year cohort of 5658 patients with hypertension in Jieshou, Anhui, China, and data from a structured household survey of a random sample (n=3005) of the cohort. The data analysis comprised (1) timeline trajectories of the rates of monthly active HBPT and mean SBP/DBP for overall and subgroups of patients with varied start-month SBP/DBP; and (2) multivariable linear, logistic, and percentile regression analyses using SBP/DBP, BP control rate, and yearly times of HBPT as the dependent variable, respectively.

**Results:**

HBPT was followed by mixed changes in mean monthly SBP/DBP for varied patient groups. The magnitude of changes ranged from –43 to +39 mmHg for SBP and from –27 to +15 mmHg for DBP. The monthly rates of active HBPT all exhibited a rapid and then gradually slower decline. When controlled for commonly reported confounders, times of HBPT in the last year were found to have decreasing correlation coefficients for SBP/DBP (from 0.16 to –0.35 and from 0.11 to –0.35, respectively) and for BP control rate (from 0.53 to –0.62).

**Conclusions:**

HBPT had major and “target-converging” effects on SBP/DBP. The magnitude of changes was much greater than commonly reported. BP, variation in BP, and time were the most important determinants of HBPT uptake. Age, education, duration of hypertension, family history, and diagnosis of hypertension complications were also linked to the uptake but at weaker strength. There is a clear need for differentiated thinking over the application and assessment of HBPT, and for identifying and correcting/leveraging potential outdated/new opportunities or beliefs.

## Introduction

Home blood pressure telemonitoring (HBPT) is recommended in current hypertension management guidelines, and is witnessing rapid diffusion worldwide [1-3]. Various randomized controlled trials (RCTs) have documented marginal to moderate effects of HBPT on blood pressure (BP), ranging from a 3 to 8 mmHg reduction in systolic blood pressure (SBP) and a 1 to 4 mmHg reduction in diastolic blood pressure (DBP) [4-6]. Studies also reported changes following HBPT in terms of quality of life, risk of cardiovascular complications, and costs due to hypertension-related service use and other outcome measures [7,8]. These effects are attributed mainly to “BP-guided” use of professional care and self-management, including self-titration of and compliance with antihypertensive medication [9,10].

Given fluctuating BP readings; changing stages (eg, normal BP, high-normal BP, grades 1 and 2 hypertension) [11] and type of hypertension (eg, office or “white coat” hypertension, masked hypertension, isolated systolic hypertension); and the varied physical, psychological, and socioeconomic conditions of patients, the actual effects of HBPT may differ greatly from patient to patient and according to the time of measurement. However, published studies on HBPT have generally adopted a “nondifferentiated” approach, focusing primarily on comparing the effects in the intervention group as a whole with those in the control group as a whole [12-14]. Although a small number of RCTs documented BP reductions for specific subgroups such as patients with inadequate baseline BP control [6,15], little is known about whether and how the effects and determinants of HBPT differ across patient groups with a varied level/stage of BP. Despite indications that the greatest effect of HBPT on BP control is usually achieved in the first months of the intervention, this is based on studies with a relatively short duration (less than 1 year) and its sustainability over the long term remains to be proven [16-18]. 

China has witnessed a rapid increase in the use of HBPT over the past decade. More and more residents are buying and using various types of HBPT devices. However, there is a general paucity of data about the effects and determinants of HBPT. Similar to studies in other countries, the limited publications on HBPT in China have focused primarily on comparing BP differences between the intervention and control groups, with little attention being paid to the determinants and differentiated effects of HBPT.

To fill this gap, the aim of this study was to use data from a relatively large-scale (5658 patients with hypertension) and long-term (up to 40 months) cohort in Jieshou, Anhui, China, for performing a relatively in-depth analysis of HBPT, with particular attention placed on comparing its effects and determinants across patient groups with varied levels of BP. As an inland county located in the middle and east of China, Jieshou is representative of the majority of counties in the nation.

## Methods

### Study Sites and Subjects

The study was built upon two related and ongoing projects. The first was initiated by Jieshou Hospital, Anhui province, China, which aimed to improve hypertension management via HBPT. The project covered all patients diagnosed with hypertension (N=5658) in all villages (N=48) served by the Jieshou Hospital Consortium. The HBPT involved an electronic oscillometric upper-arm BP monitor installed with a voice speaker capable of automatically stating the resultant measurements and educational messages to the patient. The monitors were provided by IFLYTEK Co Ltd, and were confirmed to be easily useable by ordinary residents. The readings of the HBPT were synchronously sent to a remote central data center.

The second project is an RCT registered in ISRCTN (10999269). This project used a cluster randomized sample (n=3005) of the participants in the above HBPT project to test the efficacy of a novel personalized hypertension management package [19].

By the time this study was carried out, the HBPT project had gathered BP readings from the participants for over 40 months and the RCT had completed the baseline assessment, including a structured baseline household survey.

### Data Content and Collection

This study used the records from the HBPT project described above and part of the data from the corresponding baseline household survey. Each HBPT record consisted of four items: SBP, DBP, pulse per minute, and measurement date and time. The household survey took place from April to July 2021 via a structured questionnaire administered face to face. This study used 24 items from the questionnaire, soliciting information about: (1) sociodemographic characteristics, including age, sex, and education; (2) body height and weight; (3) age when hypertension was first diagnosed; and (4) hypertension-related symptoms and diagnoses (Multimedia Appendix 1).

### Data Processing and Analysis

Data analysis comprised three components: (1) descriptive statistics (numbers and percentages) of study subjects by sociodemographic categories, (2) calculation and presentation (in trajectory lines) of the rates of monthly active HBPT for overall subjects and for subgroups with varied mean SBP/DBP in the first month, (3) multivariable linear and percentile regression modeling of times of HBPT and SBP/DBP in the last year, and (4) multivariable logistic regression modeling of BP control rate.

The rate of monthly active HBPT was defined as the proportion of patients who had performed HBPT at least one time in the month under concern. The multivariable linear, logistic, and percentile regression models used similar independent, exposure, and confounder variables. The dependent variables included times of HBPT in the past year for overall participants and subgroups with varied mean SBP/DPB from HBPT in the last year and the BP control rate in the last year. The exposure variables consisted of mean SBP/DBP and variations in the coefficients of SBP/DBP in the last year. The confounder variables comprised sociodemographics and health conditions. The monthly mean SBP/DBP of any patient was defined as their hourly mean SBP/DBP, calculated as the sum of all SBP/DBP readings recorded within a given hour (eg, 8:00-8:59 AM), multiplied by the number of records within the same hour. The BP control rate was computed as the times of BP readings meeting SBP<140 mmHg and DBP<90 mmHg in the past year multiplied by the total BP readings during the same period.

The analysis regarding the monthly active HBPT used all participants enrolled in the HBPT project, whereas the regression modeling used all of the participants involved in the baseline survey. The logarithm of times using HBPT in the last year was used to transform the variable into a normal distribution. Detailed value assignment is shown in Multimedia Appendix 1. All quantitative and ordinal variables were standardized using Z-scores before the multivariable regression modeling.

### Ethics Approval

This study has been approved by Anhui Medical University Biomedical Ethics Committee (number 20200936) and all the participants have signed (for those who are literate) or ticked (for those who are illiterate) the consent form.

## Results

### Sociodemographics of Study Participants

Of the 3005 participants recruited in the baseline survey, 57% were women. The average age of the participants was 65.50 years. Their duration of hypertension was 9.50 years on average. Over half of the respondents had a family history of hypertension (Table 1). Although detailed data about BMI and hypertension-related symptoms and diagnoses were not available for the 5658 participants in the HBPT project, they shared compatible sociodemographics with the above 3005 survey participants since the latter were a randomized sample of the former. High-normal BP formed the bulk type of hypertension (130≤SBP≤139 mmHg and/or 85≤DBP≤89 mmHg, 43.09%), followed by Grade 1 hypertension (140≤SBP≤159 mmHg and/or 90≤DBP≤99 mmHg, 32.21%) and normal BP (SBP<130 mmHg and DBP<85 mmHg, 21.37%).

**Table 1:** Sociodemographic and hypertension-related characteristics of participants (N=3005).

Variables			Sex					Total, n (%)
			Male, n (%)		Female, n (%)			
**Age (years)**								
	≤50	93 (7.20)		102 (5.95)		195 (6.49)		
	51-60	345 (26.72)		506 (29.52)		851 (28.32)		
	61-70	404 (31.29)		500 (29.17)		904 (30.08)		
	>70	449 (34.78)		606 (35.36)		1055 (35.11)		
**Education**								
	No school education	234 (18.14)		1037 (60.61)		1271 (42.35)		
	Primary school	411 (31.86)		518 (30.27)		929 (31.96)		
	Middle school or higher	645 (50.00)		156 (9.12)		801 (26.69)		
**BMI**								
	<18.5	13 (1.06)		16 (0.97)		29 (1.00)		
	1.8.5-23.9	307 (24.92)		390 (23.58)		697 (24.15)		
	24-27.9	510 (41.40)		698 (42.20)		1208 (41.86)		
	≥28	402 (32.63)		550 (33.25)		952 (32.99)		
**Duration of hypertension (years)**								
	≤4	401 (31.40)		484 (28.69)		885 (29.86)		
	5-8	320 (25.06)		433 (25.67)		753 (25.40)		
	9-12	252 (19.73)		315 (18.67)		567 (19.13)		
	>12	304 (23.81)		455 (26.97)		759 (25.61)		
**Family history of hypertension**								
	Yes	642 (54.64)		808 (51.50)		1450 (52.84)		
	No	533 (45.36)		761 (48.50)		1294 (47.16)		
**Number of hypertension-related symptoms**								
	≤4	670 (51.90)		589 (34.36)		1259 (41.90)		
	5-6	223 (17.27)		327 (19.08)		550 (18.30)		
	7-8	151 (11.70)		288 (16.80)		439 (14.61)		
	>8	247 (19.13)		510 (29.75)		757 (25.19)		
**Number of hypertension-related diagnoses**								
	0	544 (42.14)		638 (37.22)		1182 (39.33)		
	1	442 (34.24)		614 (35.82)		1056 (35.14)		
	2	223 (17.27)		312 (18.21)		535 (17.81)		
	>2	82 (6.35)		150 (8.75)		232 (7.72)		
**Type of hypertension**								
	Normal BP^a, b^	200 (19.12)		307 (23.15)		507 (21.37)		
	High-normal BP^c^	458 (43.79)		564(42.53)		1022(43.09)		
	Grade 1 hypertension^d^	346 (33.08)		418 (31.52)		764 (32.21)		
	Grade 2 hypertension^e^	42 (4.01)		37 (2.79)		79 (3.33)		
Total			1291 (43.00)		1714 (57.00)		3005 (100.00)	

^a^BP: blood pressure.

^b^Normal BP: systolic BP<130 and diastolic BP<85 mmHg.

^c^High-normal BP: 130≤systolic BP≤139 and/or 85≤diastolic BP≤89 mmHg.

^d^Grade 1 hypertension: 140≤systolic BP≤159 and/or 90≤diastolic BP≤99 mmHg.

^e^Grade 2 hypertension: systolic BP≥160 and/or diastolic BP≥100 mmHg.

### Trajectories of Monthly Mean BP Among Varied Cohorts

**Figure 1:**
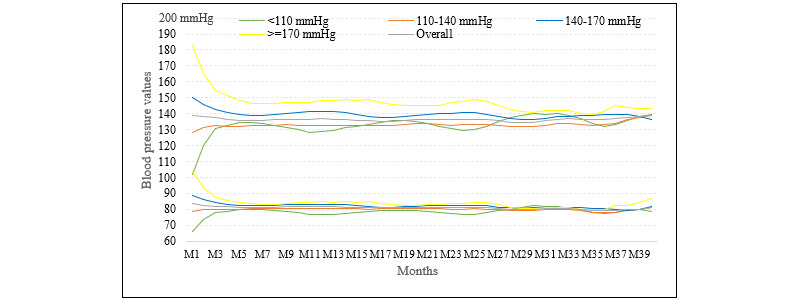
Monthly mean SBP/DBP among cohorts with varied start-month mean SBP. DBP: diastolic blood pressure; M1 through to M40: month 1 through to month 40; SBP: systolic blood pressure.

Figure 1 and Multimedia Appendix 2 demonstrate the changes in monthly mean SBP/DBP after different time periods (months) of HBPT among all 5658 participants and for patients with variable mean SBP/DBP in the first month. Both clusters of lines representing mean SBP/DBP featured a decreasing and “converging” trend, starting with a large gap between the highest and lowest mean SBP/DBP at the beginning and becoming closer and closer along the X-axis of months after the start of HBPT. The lines of mean SBP converged around a line just below 140 mmHg and the mean DBP line, just above 80 mmHg. The cohort with the highest start-month mean SBP (170+ mmHg) witnessed the greatest decrease in both SBP (from 183 mmHg in month 1 to 140 mmHg in month 35) and DBP (from 106 mmHg in month 1 to 79 mmHg in month 35). Conversely, the cohort with the lowest start-month mean SBP (110– mmHg) manifested the greatest increase in SBP (from 102 mmHg in month 1 to 141 mmHg in month 30) and DBP (from 66 mmHg in month 1 to 81 mmHg in month 40). The mean SBP/DBP among the cohort with the middle start-month mean SBP varied the least. The fastest decrease or increase occurred in the first 5-6 months.

### Rates of Monthly Active HBPT by Varied Start-Month BP

**Figure 2:**
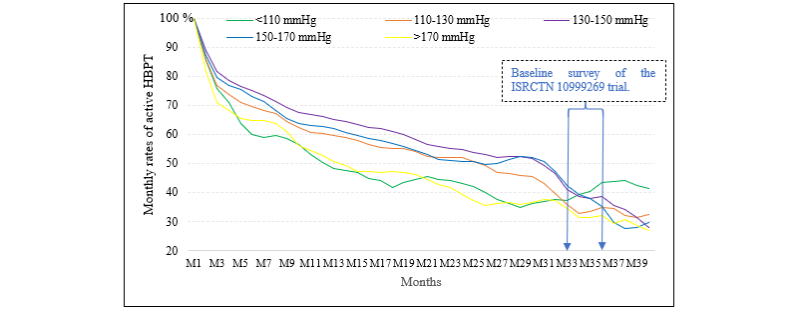
Monthly rate of active HBPT by cohorts with varied start-month systolic blood pressure. HBPT: home blood pressure telemonitoring; M1 through to M40: month 1 through to month 40.

Figure 2 presents the rates of monthly active HBPT along the time axis in months. All 5658 participants performed HBPT in the first month, but then the rates dropped quickly for the next 2 to 4 months. The rates continued to decrease at a slower and slower pace subsequently. The patients with a start-month mean SBP of 130-150 mmHg displayed the highest rate of monthly active HBPT, followed by the 150-170 mmHg and 110-130 mmHg groups. The two extreme cohorts (the 110– and 170+ mmHg groups) were the least active in terms of HBPT. When patients were grouped according to their start-month mean DBP, the trajectories of monthly active HBPT rates mimicked the results shown in Figure 2 with respect to almost all features, except for narrower gaps between different groups (Multimedia Appendix 3).

### Multivariable Regression Modeling of SBP and DBP

Table 2 summarizes the statistics of our multivariable linear and percentile regression models for mean SBP and DBP in the last year. The linear regression analysis unveiled marginal and negative relations between the times of HBPT in the last year to both SBP (B=–0.09, *P*<.001) and DBP (B=–0.11, *P*<.001). In the percentile regression models, times of HBPT were found to have decreasing correlation coefficients for the two BP variables, from 0.16 to –0.35 and from 0.11 to –0.35 for SBP and DBP, respectively. In the percentile modeling, age also showed significant associations with SBP/DBP for all percentiles (positive for SBP and negative for DBP), whereas almost no significant relations were found for education, family history, and number of hypertension-related symptoms and diagnoses to both SBP and DBP (all *P*>.05). Sex was associated with DBP but not to SBP, whereas the duration of hypertension and BMI exhibited statistically significant links on apparently more percentiles for SBP than for DBP.

**Table 2:** Multivariable linear and percentile regression modeling of mean systolic blood pressure (SBP) and diastolic blood pressure (DBP).

Variables				All patients		Percentiles of mean SBP/DBP (%)																
						10		20		30		40		50		60		70		80		90
**Systolic blood pressure**																						
	**(constant)**																					
		Correlation coefficient	—^a^		–1.14		–0.75		–0.47		–0.26		–0.04		0.20		0.46		0.73		1.21	
		*P* value	.60		<.001		<.001		<.001		<.001		.15		<.001		<.001		<.001		<.001	
	**Age**																					
		Correlation coefficient	0.20		0.19		0.20		0.18		0.17		0.16		0.17		0.19		0.21		0.24	
		*P* value	<.001		<.001		<.001		<.001		<.001		<.001		<.001		<.001		<.001		<.001	
	**Sex**																					
		Correlation coefficient	–0.05		–0.05		–0.02		–0.03		–0.06		–0.06		–0.05		–0.04		–0.07		–0.08	
		*P* value	.07		.21		.61		.27		.04		.06		.08		.25		.06		.14	
	**Education**																					
		Correlation coefficient	–0.03		0.00		–0.01		–0.04		–0.05		–0.05		–0.06		–0.05		–0.05		0.00	
		*P* value	.24		.94		.76		.17		.06		.07		.04		.10		.16		.99	
	**BMI**																					
		Correlation coefficient	0.08		0.14		0.10		0.11		0.10		0.08		0.09		0.04		0.05		0.05	
		*P* value	.001		<.001		.001		<.001		<.001		.002		<.001		.11		.12		.33	
	**Duration of hypertension**																					
		Correlation coefficient	0.10		0.12		0.10		0.07		0.08		0.08		0.09		0.10		0.10		0.11	
		*P* value	<.001		<.001		.001		.007		.002		.001		.001		<.001		.003		.03	
	**Family history of hypertension**																					
		Correlation coefficient	0.01		0.06		–0.01		–0.02		–0.02		–0.03		–0.02		0.00		0.00		0.01	
		*P* value	.65		.08		.72		.49		.49		.23		.47		.90		.94		.83	
	**Number of hypertension-related symptoms**																					
		Correlation coefficient	–0.01		0.01		–0.06		–0.06		–0.03		–0.01		0.01		0.00		0.00		0.03	
		*P* value	.60		.78		.06		.02		.24		.80		.74		.94		.99		.59	
	**Number of hypertension-related diagnoses**																					
		Correlation coefficient	–0.02		0.01		0.00		0.01		0.01		0.00		–0.03		–0.04		–0.05		–0.08	
		*P* value	.50		.67		.89		.57		.81		.98		.22		.18		.13		.11	
	**Annual measurement times**																					
		Correlation coefficient	–0.09		0.16		0.10		0.04		0.01		–0.04		–0.11		–0.18		–0.27		–0.35	
		*P* value	<.001		<.001		<.001		.10		.64		.08		<.001		<.001		<.001		<.001	
**Diastolic blood pressure**																						
	**(constant)**																					
		Correlation coefficient	—		–1.16		–0.74		–0.48		–0.22		–0.03		0.21		0.42		0.74		1.13	
		*P* value	.89		<.001		<.001		<.001		<.001		.18		<.001		<.001		<.001		<.001	
	**Age**																					
		Correlation coefficient	–0.23		–0.25		–0.23		–0.21		–0.20		–0.21		–0.24		–0.23		–0.23		–0.23	
		*P* value	<.001		<.001		<.001		<.001		<.001		<.001		<.001		<.001		<.001		<.001	
	**Sex**																					
		Correlation coefficient	–0.11		–0.10		–0.10		–0.06		–0.09		–0.07		–0.09		–0.13		–0.18		–0.16	
		*P* value	<.001		.02		.001		.04		.002		.02		.003		<.001		<.001		.002	
	**Education**																					
		Correlation coefficient	–0.02		–0.03		–0.01		0.01		0.02		0.01		0.00		–0.02		–0.06		–0.05	
		*P* value	.54		.48		.66		.79		.51		.69		.95		.57		.08		.33	
	**BMI**																					
		Correlation coefficient	0.04		0.08		0.06		0.06		0.03		0.05		0.02		0.06		0.03		0.04	
		*P* value	.08		.02		.03		.04		.20		.03		.37		.02		.26		.39	
	**Duration of hypertension**																					
		Correlation coefficient	0.03		–0.04		0.04		0.04		0.03		0.05		0.04		0.03		0.05		0.02	
		*P* value	.17		.32		.14		.12		.20		.04		.12		.19		.13		.63	
	**Family history of hypertension**																					
		Correlation coefficient	0.02		0.05		0.02		0.03		0.02		0.00		0.00		–0.04		–0.02		0.01	
		*P* value	.49		.18		.46		.25		.45		.89		.90		.17		.52		.80	
	**Number of hypertension-related symptoms**																					
		Correlation coefficient	0.00		0.05		0.01		–0.03		–0.01		–0.03		–0.01		–0.01		0.01		0.01	
		*P* value	.92		.18		.71		.31		.70		.30		.63		.81		.84		.82	
	**Number of hypertension-related diagnoses**																					
		Correlation coefficient	–0.02		0.01		0.00		–0.02		–0.03		–0.04		–0.02		–0.01		–0.04		–0.01	
		*P* value	.39		.70		.92		.49		.21		.09		.38		.60		.21		.81	
	**Annual measurement times**																					
		Correlation coefficient	–0.11		0.11		0.01		–0.04		–0.04		–0.05		–0.13		–0.17		–0.24		–0.35	
		*P* value	<.001		.003		.78		.16		.10		.06		<.001		<.001		<.001		<.001	

^a^Not applicable.

### Multivariable Logistic Regression Models of BP Control Rate

Table 3 provides statistics of nine multivariable logistic regression models of BP control rate in the last year using different cut-off values (CVs) in dividing hypertensive patients into controlled (y=1 if a patient’s BP control rate was greater than the CV) and uncontrolled (y=0 otherwise) categories. In terms of trend, times of HBPT displayed a consistent decreasing trend with BP control rate (correlation coefficient and odds ratio decreased from 0.53 and 1.7 in Model 1 to –0.62 and 0.54 in Model 9, respectively), whereas duration of hypertension presented a general increasing trend with the BP control rate from Models 1 to 9. The association of BP control rate was significant in the extreme models (Models 1, 2, 3, 4, 8, and 9) for times of HBPT, and was significant in the bottom models for age (Models 1 to 4) and number of hypertension-related diagnoses (Models 1 to 2), in top models for sex (Models 4 to 9) and BMI (from Models 7 to 9), in middle models for education (Models 3 to 5), and in all models for the duration of hypertension.

**Table 3:** Multivariable logistic regression modeling of blood pressure control rate.

Variables^a^			Model 1 (CV^b^=10%)		Model 2 (CV=20%)		Model 3 (CV=30%)		Model 4 (CV=40%)		Model 5 (CV=50%)		Model 6 (CV=60%)		Model 7 (CV=70%)		Model 8 (CV=80%)		Model 9 (CV=90%)
**(constant)**																			
	B^c^	1.25		0.61		0.27		–0.05		–0.35		–0.76		–1.24		–1.63		–2.49	
	OR^d^	3.50		1.84		1.30		0.95		0.71		0.47		0.29		0.20		0.08	
	*P* value	<.001		<.001		<.001		.30		<.001		<.001		<.001		<.001		<.001	
**Age**																			
	B	0.17		0.19		0.19		0.12		0.08		0.03		–0.04		–0.05		–0.06	
	OR	1.19		1.20		1.21		1.13		1.09		1.03		0.96		0.95		0.94	
	*P* value	.007		.001		<.001		.02		.13		.55		.50		.49		.49	
**Sex**																			
	B	0.12		0.09		0.09		0.14		0.11		0.16		0.19		0.18		0.20	
	OR	1.13		1.09		1.10		1.15		1.12		1.17		1.21		1.19		1.22	
	*P* value	.08		.14		.10		.01		.05		.01		.005		.02		.04	
**Education**																			
	B	0.11		0.07		0.11		0.12		0.11		0.09		0.12		0.14		0.12	
	OR	1.11		1.07		1.12		1.13		1.12		1.09		1.13		1.15		1.13	
	*P* value	.12		.25		.05		.03		.05		.14		.08		.06		.22	
**BMI**																			
	B	0.04		0.00		-0.01		–0.04		–0.06		–0.09		–0.14		–0.14		–0.17	
	OR	1.04		1.00		0.99		0.96		0.94		0.92		0.87		0.87		0.84	
	*P* value	.50		.94		.85		.37		.21		.10		.02		.03		.05	
**Duration of hypertension**																			
	B	–0.19		–0.13		–0.11		–0.12		–0.16		–0.21		–0.25		–0.21		–0.31	
	OR	0.83		0.87		0.89		0.88		0.86		0.81		0.78		0.81		0.73	
	*P* value	.001		.007		.02		.01		.002		<.001		<.001		.004		.003	
**Family history of hypertension**																			
	B	0.02		0.02		0.01		-0.02		0.05		0.08		0.03		0.00		–0.04	
	OR	1.02		1.02		1.01		0.98		1.05		1.09		1.03		1.00		0.96	
	*P* value	.67		.74		.81		.70		.29		.11		.58		.97		.61	
**Number of hypertension-related symptoms**																			
	B	0.03		–0.02		–0.01		–0.02		–0.02		–0.01		–0.02		0.01		–0.08	
	OR	1.03		0.98		0.99		0.98		0.98		0.99		0.98		1.01		0.92	
	*P* value	.61		.68		.86		.65		.69		.89		.69		.92		.38	
**Number of hypertension-related diagnoses**																			
	B	0.15		0.10		0.09		0.10		0.02		–0.03		–0.01		–0.06		0.04	
	OR	1.16		1.11		1.09		1.10		1.02		0.97		0.99		0.94		1.04	
	*P* value	.01		.04		.08		.05		.63		.51		.88		.36		.64	
**Annual measurement times**																			
	B	0.53		0.24		0.15		0.12		0.03		0.02		–0.09		–0.22		–0.62	
	OR	1.70		1.27		1.16		1.12		1.03		1.02		0.92		0.80		0.54	
	*P* value	<.001		<.001		.002		.02		.58		.75		.12		<.001		<.001	

^a^The dependent variable in Models 1 to 9 was assigned 1 if the blood control rate of the patient under concern was greater than the CV or 0 otherwise.

^b^CV: cut-off value of blood pressure control rate.

^c^B: correlation coefficient.

^d^OR: odds ratio.

### Multivariable Percentile Regression Analysis of HBPT

**Figure 3:**
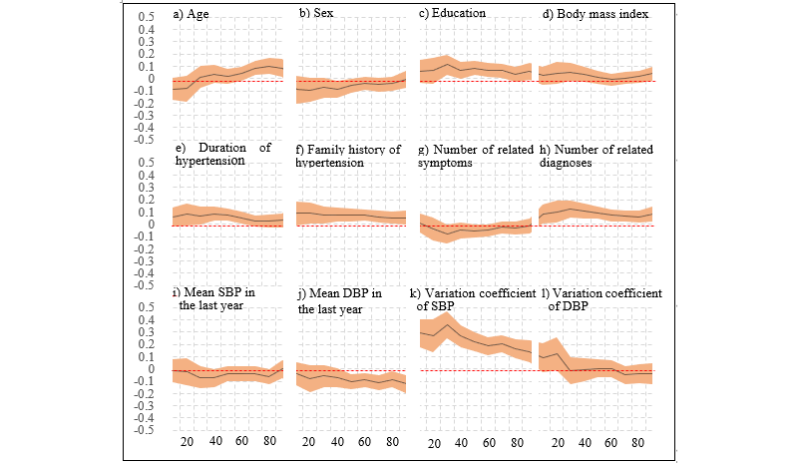
Multivariable percentile regression modeling of factors affecting times of home blood pressure telemonitoring (HBPT). The y-axis represents the regression coefficient. The x-axis represents quantiles of times of HBPT in the last year. DBP: diastolic blood pressure; SBP: systolic blood pressure.

Figure 3 displays, in shaded curves, the multivariable percentile regression coefficients between times of HBPT in the last year and the independent variables studied. Of all the curves, only those representing the variation coefficients of SBP (Figure 3k) and number of hypertension-related diagnoses (Figure 3h) presented a clear distance from the dashed red line (B=0) along all of the percentiles, and only the curve representing the mean SBP overlapped with the red line along the entire percentile axis (Figure 3i). All of the remaining shaded curves demonstrated interceptions with the red line for a larger or smaller part of the percentiles. The variation coefficients of SBP (Figure 3k) manifested the largest distance from the red line and exhibited a decreasing trend along the percentiles, whereas the coefficient of age presented an increasing trend. The main statistics of the multivariable percentile regression model are given in Multimedia Appendix 4. 

## Discussion

### Effects of HBPT on SBP/DBP

Our study unveiled novel and meaningful BP trajectories after HBPT among hypertensive cohorts with varied mean SBP in the first month (Figure 1). Instead of simply lowering SBP or DBP as documented in most previous related studies, HBPT followed mixed changes in our study depending on the resultant B*P* values of the patients under concern. The magnitude of changes ranged from –43 to +39 mmHg for SBP and from –27 to +15 mmHg for DBP. When controlled for commonly reported confounder variables such as sex, age, education, duration of hypertension, BMI, family history of hypertension, and numbers of hypertension-related symptoms and diagnoses, the differentiated effects of HBPT on SBP/DBP were still observable. In the multivariable percentile regression model (Table 2), times of HBPT showed moderate to strong relations with both SBP and DBP. In our multivariable logistic regression models (Table 3), times of HBPT again demonstrated strong and differentiated associations with BP control rate. These findings suggest that HBPT played major yet bidirectional or “target-converging” roles. Interestingly, the “target” here was the widely validated and accepted defining values of hypertension control (ie, SBP below 140 and/or DBP under 90 mmHg [11,20]). When the monitored SBP/DBP was higher than the target, HBPT may have urged the patients to take actions to reduce their BP through self-titrating antihypertensive medication; consulting their doctors for initiating or intensifying antihypertensive treatment; and practicing more rigorous lifestyle changes that have been shown to reduce hypertension, including weight loss, dietary approaches, and physical activity [21-24]. For patients with lower than the target BP, HBPT may have informed them to consult their doctors for milder treatment agents or doses, or to perhaps reduce further self-management efforts.

Implications of the “target-converging” effect remain to be carefully examined. It is well-established that “convergence” downward from above the “target” is beneficial to patients via various mechanisms, including a lower risk of cerebral hemorrhage [25,26]. An upward “convergence” from far below the “target” (eg, SBP<110 mmHg) may also result in better health outcomes due to, for instance, reduced chances of cerebral ischemia [27]. However, upward “convergence” from a certain range below the “target” (eg, SBP from 130 mmHg to 140 mmHg) may be harmful to patients’ health.

### Determinants of HBPT

The declining and varied rates of monthly active HBPT for different cohorts (Figure 2) suggest that SBP/DBP and time may be the most important factors affecting HBPT. The reasons why the middle cohort (subgroups with a start-month mean SBP=130-150 mmHg in Figure 2 or DBP=80-90 mmHg in Multimedia Appendix 3) exhibited the highest rates of monthly HBPT may be because their resultant SBP/DBP levels were the closest to the defining values of hypertension control. The closer a patient’s BP is to the defining value, the higher their chances to obtain meaningful feedback (success or failure in hypertension management) from an HBPT, and thus the greater the desire to perform the monitoring. Conversely, participants in the two extreme cohorts may have become either frustrated with or relieved to perform HBPT.

The decreasing trend over time following start of the HBPT project may be mainly attributed to increasing familiarity with the resultant SBP/DBP. In other words, when the patients’ ability to anticipate the results enhanced, their desire or interest in performing HBPT decreased. This is consistent with our findings (Figure 3 and Multimedia Appendix 4) that the variation coefficients of SBP were independently linked to the times of HBPT.

Our multivariable percentile regression model also identified independent associations between HBPT and age, education, duration of hypertension, family history, and diagnosis of hypertension complications. Perceived risk may be the main reason underlying these relations. In other words, patients of older age, with better education, a longer duration of hypertension, more diagnoses, and family history may perceive themselves at an elevated risk for developing hypertension complications and thus become more active in HBPT [28,29]. It is worth noting that all of these correlations were weaker than those of the values of DBP and variations in SBP in terms of the magnitude of the correlation coefficient or the duration of percentiles.

### Variations in Relationships

Our study uncovered interesting variations in the relationships between HBPT and its influencing factors. Times of HBPT presented negative associations with mean DBP (Figure 3j), but did not show statistically significant associations with mean SBP (Figure 3i). This may be explained by a dynamic interaction between the dependent and independent variables. More specifically, more frequent HBPT led to greater chances for identifying elevated BP, which in turn led to greater efforts to reduce BP and then to greater decreases in BP, and finally to nonsignificant relations between SBP and HBPT. The same dynamics could also be in play for DBP but led to negative associations, since a substantial portion of the patients had isolated systolic hypertension and their DBP was indirectly reduced via the interactions between HBPT and SBP. The variation in SBP (Figure 3k) could not be as easily reduced as SBP/DBP via the interaction dynamics, and thus showed consistent strong correlations with HBPT. The nonsignificant relations between variation in DBP and HBPT (Figure 3l) may be related to the much smaller value as compared with the variation in SBP, which thus attracted relatively little attention from the patients.

Similar variations in correlations were also observed in the models using SBP/DBP as the dependent variables. For example, age showed a sustained and positive association with SBP but a continuous negative link to DBP. These contradictory relations have been reported in various hypertensive populations, especially those dominated by relatively older patients with isolated systolic hypertension [30,31]. In addition, sex was associated with DBP but not SBP, while BMI and duration of hypertension showed stronger links with SBP than with DBP. These findings are also similar with those of previous studies [32,33]. With regard to the BP control rate, our logistic regression model suggested that age, sex, and education were protective factors; BMI and number of hypertension-related diagnoses were risk factors; and the effects of these factors were complex, being observable in various parts of the models. The mechanisms and implications of these phenomena merit further exploration.

### Strengths and Limitations

Our study has both strengths and limitations. This study used data from a relatively large-scale (5658 patients with hypertension) and long-term cohort. Relatively in-depth analysis of the determinants of HBPT was performed, with particular attention paid to subtle and differential interactions with the resultant BP outcome. This study thus produced useful trajectories of monthly mean SBP/DBP and monthly active rates of HBPT for up to 40 months. Multivariable linear, percentile, and logistic regression modeling of times of HBPT, mean SBP/DBP, and BP control rate as the dependent variables, respectively, enabled cross-checks and comparisons of the results.

This study also suffers from drawbacks. First, being performed at home by ordinary residents, the B*P* values from HBPT are prone to various influences. Second, the study population was relatively old (65.50 years on average) and the findings should be generalized with caution. Third, the study considered only SBP/DBP as the outcome variables without considering others (eg, complications, health care burden) and lacked comparison with patients who had not used HBPT. Fourth, BP readings are susceptible to diurnal and intraobserver variations, which can lead to measurement biases, although our use of monthly and hourly mean SBP/DBP may have helped to reduce these biases to some extent. Our further research activities in response to these shortcomings include performing household surveys/observations to help identify the factors influencing HBPT readings, extend the HBPT to younger populations, and perform further analyses linking HBPT with major adverse cardiovascular events (eg, apoplexy) and quality of life.

### Conclusions

HBPT had major and “target-converging” effects on SBP/DBP. The “target” was the widely validated and accepted defining values of hypertension control (ie, SBP below 140 and/or DBP under 90 mmHg). HBPT was followed by SBP/DBP reductions or increases for cohorts with a mean BP higher or lower than the “target,” respectively. The magnitude of changes was a few times greater than commonly documented. These differentiated effects remained observable into the third year after initiation of HBPT. BP, variation in BP, and time were the most important determinants of HBPT uptake, whereas age, education, duration of hypertension, family history, and diagnosis of hypertension complications were also linked to the uptake but at apparently weaker strength. HBPT displayed stronger associations with the variation in SBP than in DBP.

There is a clear need for differentiated thinking over the application and assessment of HBPT. First, the traditional approach of simply comparing the effects in the intervention group as a whole with that in the control group is prone to underestimation of the actual influences of HBPT, since decreases in a portion of the patients were offset by increases in others. HBPT leads to BP decrease, stability, or increase depending on the complex and dynamic context of the patient under concern. These varied effects may not necessarily all be beneficial and merit careful scrutiny in the future. This study thus highlights the need for correcting outdated beliefs or practices and leveraging new opportunities with the application of HBPT. Second, the difference in the “white coat” effect suggests lower than traditional cut-off values of hypertension control when readings from HBPT were used. In other words, patients should be better educated about the “white coat” effect and that they need to exert further efforts to maintain their HBPT readings slightly below 140/90 mmHg. Third, the varied responses toward different levels of HBPT readings indicate selective telemonitoring, group-specific “targets,” or even personalized interventions. Fourth, relatively less attention paid to DBP than to SBP implies that additional efforts are needed to promote balanced awareness among patients. In particular, patients should be informed that DBP is as important as SBP and thus merits equal attention in self-monitoring.

## Appendix

Multimedia Appendix 1 Related questions used in the baseline household survey and value assignment.

Multimedia Appendix 2 Monthly mean SBP/DBP among cohorts with varied start-month mean DBP. DBP: diastolic blood pressure; SBP: systolic blood pressure; M1 through to M40: month 1 through to month 40.

Multimedia Appendix 3 Monthly rate of active HBPT by cohorts with varied start-month diastolic blood pressure. HBPT: home blood pressure telemonitoring; M1 through to M40: month 1 through to month 40.

Multimedia Appendix 4 Multivariable linear and percentile regression coefficients of times of home blood pressure telemonitoring (HBPT) .

## Abbreviations

BP: blood pressure
CV: cut-off value
DBP: diastolic blood pressure
HBPT: home blood pressure telemonitoring
RCT: randomized controlled trial
SBP: systolic blood pressure
