# Supplementary material for: Differentiated Effects and Determinants of Home Blood Pressure Telemonitoring: Three-Year Cohort Study in Jieshou, Anhui, China
Source: J Med Internet Res. 2022 Oct 11;24(10):e37648. doi: 10.2196/37648 (PMC9597421; doi:10.2196/37648)
Supplement: Multimedia Appendix 1 [file jmir_v24i10e37648_app1.docx]

**Multimedia Appendix 1**

**Related questions used in the baseline household survey and value assignment**

Part A: Sociodemographic characteristics

Q1: Enter the respondent’s sex.

[ ] Male(X1=0)

[ ] Female(X1=1)

Q2: Measure the respondent’s height.

[ ] m(X2=value entered)

Q3: Measure the respondent’s weight.

[ ] kg(X3=value entered)

Q4: How old are you?

[ ] years(X4=value entered)

Q5: What’s your highest education?

[ ] No school education(X5=1)

[ ] Primary school(X5=2)

[ ] Middle school or higher(X6=3)

Q6: When were you diagnosed with hypertension?

[ ] years(X6=value entered)

Q7: Has any of your family members or relatives had high blood pressure?

[ ] No(X7=0)

[ ] Yes(X7=1)

Part B: Complications

Q8: (hypertension related symptoms): Have you felt or experienced the following symptoms during the past month? (Please tick **all** items that suit you)

[ ] Headache or dizziness (If ticked X8a=1,otherwise X8a=0)

[ ] Dazzling lights or black out (If ticked X8b=1,otherwise X8b=0)

[ ] Tinnitus or syrigmus (If ticked X8c=1,otherwise X8c=0)

[ ] Heartache or chest ache (If ticked X8d=1,otherwise X8d=0)

[ ] Palpitation (If ticked X8e=1,otherwise X8e=0)

[ ] Memory loss (If ticked X8f=1,otherwise X8f=0)

[ ] Inattention (If ticked X8g=1,otherwise X8g=0)

[ ] insomnia or dreaminess (If ticked X8h=1,otherwise X8h=0)

[ ] Easily excited and fidgety (If ticked X8i=1,otherwise X8i=0)

[ ] Limb numbness (If ticked X8j=1,otherwise X8j=0)

[ ] Edema of eyelid (If ticked X8k=1,otherwise X8k=0)

[ ] Edema of lower extremities (If ticked X8l=1,otherwise X8l=0)

(X8=X8a+X8b+X8c+X8d+X8e+X8f+X8g+X8h+X8i+X8j+X8k+X8l)

Q9: (hypertension related diagnosis): Have you ever been diagnosed by a doctor the following? (Please tick **all** items that suit you)

[ ] Myocardial infarction (If ticked X9a=1,otherwise X9a=0)

[ ] Cerebral infarction (If ticked X9b=1,otherwise X9b=0)

[ ] Atherosclerosis (If ticked X9c=1,otherwise X9c=0)

[ ] Impaired renal function (If ticked X9d=1,otherwise X9d=0)

(X9=X9a+X9b+X9c+X9d)
