# Supplementary figures and images for: Differentiated Effects and Determinants of Home Blood Pressure Telemonitoring: Three-Year Cohort Study in Jieshou, Anhui, China
Source: J Med Internet Res. 2022 Oct 11;24(10):e37648. doi: 10.2196/37648 (PMC9597421; doi:10.2196/37648)

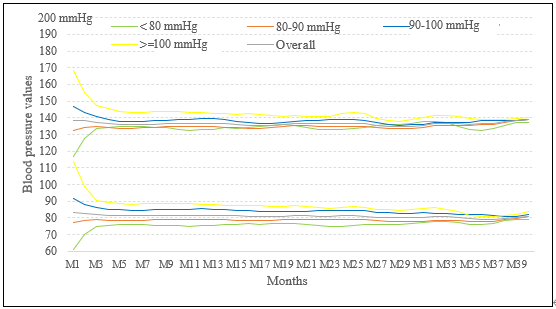

Supplement: Multimedia Appendix 2 [file jmir_v24i10e37648_app2.png]

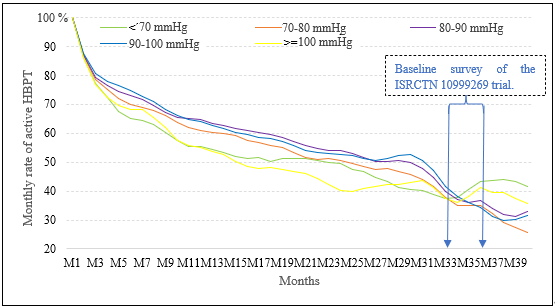

Supplement: Multimedia Appendix 3 [file jmir_v24i10e37648_app3.png]
