# Supplementary material for: Differentiated Effects and Determinants of Home Blood Pressure Telemonitoring: Three-Year Cohort Study in Jieshou, Anhui, China
Source: J Med Internet Res. 2022 Oct 11;24(10):e37648. doi: 10.2196/37648 (PMC9597421; doi:10.2196/37648)
Supplement: Multimedia Appendix 4 [file jmir_v24i10e37648_app4.docx]

**Multimedia Appendix 4**

**Multivariable linear and percentile regression coefficients of times of home blood pressure telemonitoring (HBPT)**

| **Variables** | **All patients** | **Percentiles of HBPT (%)** | | | | | | | | |  |
| --- | --- | --- | --- | --- | --- | --- | --- | --- | --- | --- | --- |
|  |  | **10** | **20** | **30** | **40** | **50** | **60** | **70** | **80** | **90** |  |
| （constant） | — | -1.53 | -0.99 | -0.56 | -0.28 | -0.05 | 0.17 | 0.40 | 0.67 | 1.03 |  |
| *P* | .291 | <.001 | <.001 | <.001 | <.001 | .268 | <.001 | <.001 | <.001 | <.001 |  |
| Age | 0.02 | -0.08 | -0.08 | 0.01 | 0.04 | 0.02 | 0.04 | 0.09 | 0.10 | 0.09 |  |
| *P* | .512 | .076 | .136 | .777 | .320 | .515 | .172 | .003 | .001 | .016 |  |
| Sex | -0.06 | -0.09 | -0.09 | -0.08 | -0.09 | -0.06 | -0.04 | -0.05 | -0.04 | -0.01 |  |
| *P* | .018 | .115 | .078 | .065 | .007 | .054 | .190 | .085 | .123 | .933 |  |
| Education | 0.06 | 0.06 | 0.06 | 0.11 | 0.07 | 0.01 | 0.07 | 0.06 | 0.03 | 0.06 |  |
| *P* | .025 | .200 | .226 | .009 | .051 | .003 | .012 | .023 | .278 | .098 |  |
| Body mass index (BMI) | 0.02 | 0.02 | 0.05 | 0.05 | 0.03 | 0.01 | -0.01 | 0.00 | 0.01 | 0.04 |  |
| *P* | .396 | .569 | .315 | .143 | .278 | .671 | .687 | .891 | .579 | .181 |  |
| Duration of hypertension | 0.05 | 0.06 | 0.08 | 0.07 | 0.09 | 0.08 | 0.05 | 0.03 | 0.03 | 0.03 |  |
| *P* | .018 | .137 | .082 | .068 | .005 | .002 | .028 | .293 | .315 | .290 |  |
| Family history of hypertension | 0.07 | 0.09 | 0.09 | 0.07 | 0.08 | 0.08 | 0.08 | 0.06 | 0.05 | 0.05 |  |
| *P* | .001 | .077 | .035 | .042 | .010 | .001 | <.001 | .005 | .059 | .081 |  |
| Number of related symptoms ^a^ | -0.03 | 0.01 | -0.04 | -0.08 | -0.05 | -0.06 | -0.05 | -0.03 | -0.03 | -0.02 |  |
| *P* | .171 | .827 | .401 | .043 | .108 | .034 | .049 | .321 | .316 | .604 |  |
| Number of related diagnosis ^b^ | 0.09 | 0.09 | 0.10 | 0.12 | 0.11 | 0.09 | 0.07 | 0.07 | 0.06 | 0.08 |  |
| *P* | <.001 | .029 | .024 | .001 | .001 | <.001 | .003 | .007 | .019 | .005 |  |
| Mean SBP ^c^ in the last year | -0.04 | -0.02 | -0.03 | -0.07 | -0.07 | -0.04 | -0.04 | -0.04 | -0.07 | 0.00 |  |
| *P* | .132 | .726 | .646 | .127 | .048 | .174 | .168 | .206 | .043 | .962 |  |
| Mean DBP ^d^ in the last year | -0.08 | -0.04 | -0.08 | -0.06 | -0.07 | -0.10 | -0.09 | -0.11 | -0.09 | -0.12 |  |
| *P* | .005 | .423 | .147 | .218 | .056 | .001 | .003 | <.001 | .010 | .001 |  |
| VC ^e^ of SBP in the last year | 0.23 | 0.29 | 0.27 | 0.36 | 0.27 | 0.22 | 0.19 | 0.21 | 0.16 | 0.14 |  |
| *P* | <.001 | <.001 | <.001 | <.001 | <.001 | <.001 | <.001 | <.001 | <.001 | .002 |  |
| VC of DBP in the last year | 0.05 | 0.09 | 0.12 | -0.02 | -0.01 | 0.00 | 0.00 | -0.05 | -0.04 | -0.04 |  |
| *P* | .189 | .118 | .067 | .770 | .895 | .919 | .964 | .162 | .295 | .377 |  |
| Note: ^a, b^ related symptoms or diagnosis means hypertension-related symptoms/diagnosis; ^c^ SBP=systolic blood pressure; ^d^ DBP=diastolic blood pressure; ^e^ VC=variation coefficient; the lines above the *P* values represent the correlation coefficients for the independent variables. | | | | | | | | | | | |
